# Degradation Monitoring of Insulation Systems Used in Low-Voltage Electromagnetic Coils under Thermal Loading Conditions from a Creep Point of View

**DOI:** 10.3390/s20133696

**Published:** 2020-07-01

**Authors:** Kai Wang, Haifeng Guo, Aidong Xu, Michael Pecht

**Affiliations:** 1 Key Laboratory of Networked Control Systems, Chinese Academy of Sciences, Shenyang 110016, China; guohaifeng@sia.cn (H.G.); xad@sia.cn (A.X.); 2 Shenyang Institute of Automation, Chinese Academy of Sciences, Shenyang 110016, China; 3 Institutes for Robotics and Intelligent Manufacturing, Chinese Academy of Sciences, Shenyang 110169, China; 4 University of Chinese Academy of Sciences, Beijing 100049, China; 5 Liaoning Institute of Science and Technology, Benxi 117004, China; 6 Center for Advanced Life Cycle Engineering, University of Maryland, College Park, MD 20742, USA; pecht@umd.edu

**Keywords:** electromagnetic coil, insulation degradation monitoring, high-frequency electrical parameter, creep deformation, accelerated testing

## Abstract

Electromagnetic coils are a key component in a variety of systems and are widely used in many industries. Because their insulation usually fails suddenly and can have catastrophic effects, degradation monitoring of coil insulation systems plays a vital role in avoiding unexpected machine shutdown. The existing insulation degradation monitoring methods are based on assessing the change of coil high-frequency electrical parameter response, whereas the effects of the insulation failure mechanisms are not considered, which leads to inconsistency between experimental results. Therefore, this paper investigates degradation monitoring of coil insulation systems under thermal loading conditions from a creep point of view. Inter-turn insulation creep deformation is identified as a quantitative index to manifest insulation degradation changes at the micro level. A method is developed to map coil high-frequency electrical monitoring parameters to inter-turn insulation creep deformation in order to bridge the gap between the micro-level and macro-level changes during the incipient insulation degradation process. Thermally accelerated tests are performed to validate the developed method. The mapping method helps to determine the physical meaning of coil electrical monitoring parameters and presents opportunities for predictive maintenance of machines that incorporate electromagnetic coils.

## 1. Introduction

Electromagnetic coils are fundamental energy conversion and transformation components of many systems [1]. Although they are widely used in motors, transformers, and solenoids, they are failure-prone. Many papers have reported that stator-winding insulation is one of the weakest components in an electric machine [2,3,4,5]. A study conducted by Oak Ridge National Laboratory [6] showed that over 50% of solenoid valve failures in U.S. nuclear power plants were attributed to electromagnetic coil faults (e.g., coil open, coil short). For electric generators, 56% of failures originated as electrical insulation damage [7,8]. Further, insulation failure is one of the dominant failure modes for classical wind turbines [9]. New applications, especially in more-electric aircraft, should widen the use of low-voltage (under 1 kV) electrical rotating machines [10]. However, approximately 40% of the failures in low-voltage rotating machines have originated from the stator-winding insulation materials [11]. The reliability of the low-voltage coil insulation systems in rotating machines has thus become a critical issue and requires technical-conditions-monitoring to avoid unexpected shutdown of machines that incorporate electromagnetic coils.

**Figure 1:**
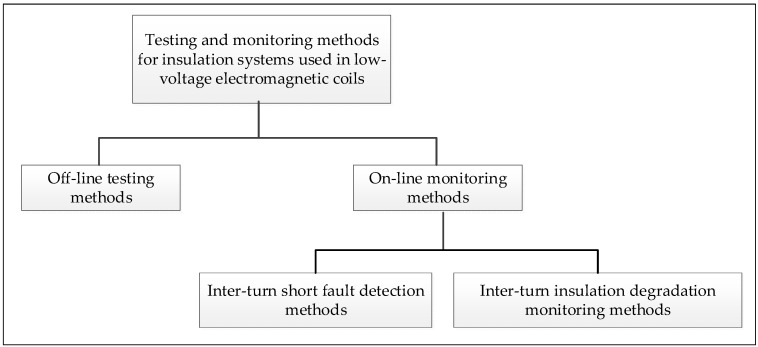
Categories for testing and monitoring methods for insulation systems used in low-voltage electromagnetic coils.

Testing and monitoring methods for insulation systems of electromagnetic coils have been surveyed in Grubic et al. [12]. The classical methods used to characterize the quality of a winding insulation can be categorized into off-line testing and on-line monitoring methods, which are shown in Figure 1. The off-line testing methods are so far the most industrially accepted [13] and are usually based on visual inspection and electrical, physical, and chemical measurement. For example, Stone [14,15] discussed a few methods that rely on physical contact with and measurement of the insulation (e.g., capacitance, dissipation factor, and insulation resistance) as a dielectric between the wire conductor and an outside ground plane. In this case, electrodes are required to be placed outside the insulation. Considering that electromagnetic coils in solenoid valves or motors are generally encased or otherwise unavailable for intrusive inspection, these methods require the machines under test to stop and to be disassembled, which means that they can only be applied off-line during predefined maintenance periods. Because off-line testing methods thus interrupt the running of the plant, continuous health assessment of the coils through on-line monitoring is preferred to improve the plant safety levels and optimize the maintenance schedule.

**Figure 2:**
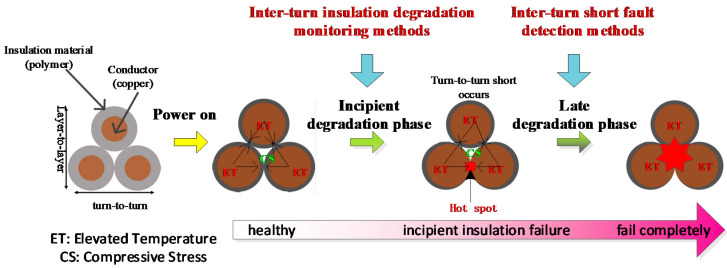
Incipient and late degradation phases of electromagnetic coils.

The on-line monitoring methods can be further divided into inter-turn short fault detection methods and inter-turn insulation degradation monitoring methods. In order to illustrate the relationship between these two methods clearly, insulation deterioration process is analyzed, as shown in Figure 2. Since insulation breakdown of electromagnetic coils usually originates from a turn-to-turn short [16], the deterioration process is divided into incipient and late degradation phases according to whether or not the turn-to-turn fault has occurred [17]:

➢ **Incipient degradation phase:** As a current is passed through the wire, Joule heating causes an increase in the wire temperature, which results in expansion of the conductor (usually copper) and thus compressive stress on turn-to-turn insulation (usually a polymer). Under the long-term effects of compressive and thermal stresses on the turn-to-turn insulation, the insulation materials degrade and lead to a turn-to-turn short.
➢ **Late degradation phase:** After the turn-to-turn short occurs, the direct-current (DC) resistance of the coil decreases, and thus current increases, which makes the coil temperature increase. A hot spot can form at the location of the short. Turn-to-turn short faults will spread quickly around the hot spot, which causes the coil to burn out, finally resulting in failure of the complete coil.

Accordingly, the inter-turn short fault detection methods are used in late degradation phase to detect if the turn-to-turn short forms or to assess fault severity by identifying the number of inter-turn shorts, whereas the inter-turn insulation degradation monitoring methods are used in the incipient degradation phase to monitor the health state of insulation before the turn-to-turn short formation.

**(1) Inter-turn short fault detection methods**

Motor current signature analysis (MCSA) is commonly used to detect inter-turn short fault by analyzing the stator current spectrum. The basic principle of MCSA is that the frequency domain features of the stator current will change (e.g., 3rd harmonic was observed) in the event of an inter-turn short. The classical MCSA methods include Fourier-transform-based methods [18,19], and wavelet-transform-based methods [20,21].

Other commonly used methods are based on the principle that motor symmetry is changed after the turn-to-turn short formation. The classical methods include the negative-sequence-current-component-based methods [22], Park’s vector approach, and extended Park’s-vector-based methods [23].

Artificial-intelligence-based methods are proposed in order to eliminate the interference of noise during fault detection, in which winding current signals with different health states are acquired to train classifiers. Thus, these methods can be used to classify the different short circuit levels by identifying the number of inter-turn shorts. The most common methods are based on artificial neural network (ANN) [24] and support vector machine (SVM) [25].

Motor magnetic field signals can also be used to detect inter-turn shorts, wherein the motor magnetic field changes in the event of an inter-turn short fault. The classical methods include air-gap-flux-based methods [26,27] by installing search coils inside the motor and leakage-flux-based approaches [28,29] using an external flux sensor outside the motor. Motor magnetic-field-signal-based methods can provide fault location information comparing to the MCSA-based methods.

According to the analysis mentioned above, the application premise of inter-turn short detection methods is that the turn-to-turn short has already formed. However, once the turn-to-turn short forms, the coil insulation degradation process accelerates, and the impending insulation failure usually occurs suddenly and has catastrophic effects. For example, it has been shown that it takes less than 2 s for a single turn fault to develop into a critical fault in a typical 15-kW induction motor [30]. Therefore, inter-turn short fault detection methods have limited significance for predictive maintenance of machines that incorporate coils.

**(2) Inter-turn insulation degradation monitoring methods**

Considering coil insulation systems usually go through a slow degradation process before a turn-to-turn fault occurs, inter-turn insulation degradation monitoring presents opportunities for continuous technical conditions monitoring and predictive maintenance of machines that incorporate electromagnetic coils. The representative inter-turn insulation degradation monitoring methods include Werynski et al. [31], who performed an accelerated aging test on twisted pairs of magnet wire with polyester-imide insulation and found that the insulation capacitance increased as breakdown voltage decreased. The phase shift between a signal injected into a motorette, which is designed to be a small-scale model of electric motor stator windings, at the coil resonant frequency and the resulting magnetic field were used as the health indicator for the insulation. Perisse et al. [32,33] showed that changes in the turn insulation capacitance of the electromagnetic coil are reflected in the resonant frequency and thus developed a monitoring system able to detect slight variations of high-frequency resonances in the winding of a working machine fed by an industrial inverter. Savin et al. [34,35] also placed twisted pairs of magnet wire with layered polyester-imide/polyester-amide insulation under thermal stress and found that the partial discharge inception voltage decreased, which is an indication of insulation degradation, whereas the turn-to-turn capacitance increased. Thus, they claimed that the capacitance can be used as an aging indicator of the winding insulation. Zoeller et al. [36,37,38] proposed a method for stator insulation defect detection in traction-drive machine windings by evaluating the current response after a voltage step excitation, which is based on the fact that parasitic winding capacitance changes as the insulation degrades. Nussbaumer et al. [13] proposed a method to monitor changes in the insulation health state by evaluating high-frequency machine properties. The insulation state indicator was defined by feature extraction of the high-frequency information. Jameson et al. [1,39] proposed a low-voltage coil insulation health monitoring method based on the impedance spectrum. The Spearman correlation coefficient was used to find frequency regions of interest within the impedance spectrum.

In summary, the existing inter-turn insulation degradation monitoring methods detect degradation of insulation used in low-voltage coils by assessing the change of high-frequency electrical parameters (e.g., impedance, resonant frequency, and capacitance) response, whereas the effects of insulation failure mechanism on these electrical parameters were not considered. This brings inconsistency between the experimental results of existing studies. Some representative examples are as follows.

(1) There are contradictory claims for the evolving trends of high-frequency electrical parameters in the literature. For example, Perisse et al. [32] and Savin et al. [34] claimed that the capacitance increased over the aging period while Younsi et al. [40] and Zhang et al. [41] found that the capacitance decreased during the aging time.
(2) Perisse et al. [33] set a failure threshold of 95% of the healthy coil resonant frequency and mentioned that once the failure threshold is crossed, the coil should be maintained under monitoring. However, the experimental results in Jameson et al. [42] showed that the resonant frequency changed by only 2.5% before a short formed.

**Figure 3:**
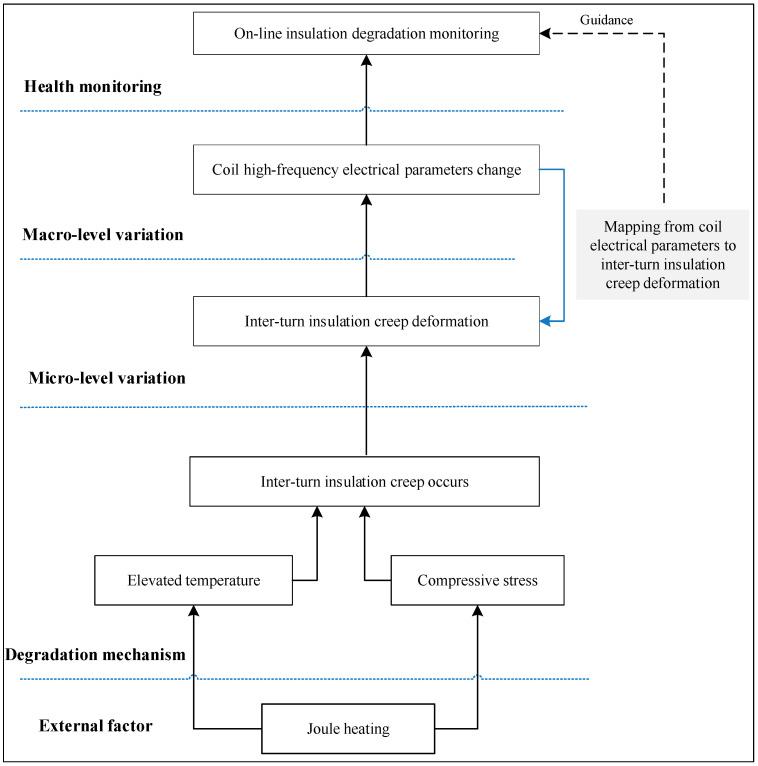
Framework for coil insulation degradation monitoring from a creep point of view developed in this paper.

The difference between the results from experiments in the previous studies may be due to differences in material properties and failure mechanisms. It is premature to make a conclusion on electrical parameter evolving trends or setting failure thresholds without considering insulation failure mechanisms. It is thus important to illustrate the insulation degradation mechanism, examine related material-level changes under certain loading conditions, and associate those changes with evolving electrical parameters when performing on-line degradation monitoring in situ. Therefore, this paper has developed a creep degradation mechanism for inter-turn insulation, aiming at thermal loading conditions that have been shown to be the primary reason for insulation failure [43]. The framework for insulation degradation monitoring from a creep point of view is shown in Figure 3. On the one hand, creep deformation of inter-turn insulation is the manifestation of insulation degradation on the micro level and can be used to assess the insulation’s health state. However, creep deformation is not easy to measure in-site. On the other hand, coil high-frequency electrical parameters are the manifestation of insulation degradation on the macro level and can be measured in-site. Therefore, a mapping method from coil high-frequency electrical parameters to inter-turn insulation creep deformation is developed, which associates changes at the micro level and macro level during insulation degradation.

The remainder of this paper is organized as follows. Section 2 analyzes the creep degradation mechanism of inter-turn insulation systems and its effects on coil high-frequency electrical parameter response. Section 3 presents a mapping method from the coil electrical parameters to inter-turn insulation creep deformation. Section 4 presents the experimental setup and results. Section 5 concludes the paper.

## 2. Coil Creep Degradation Mechanism Analysis

### 2.1. Creep Degradation of Inter-Turn Insulation Systems under Thermal Loading Conditions

**Figure 4:**
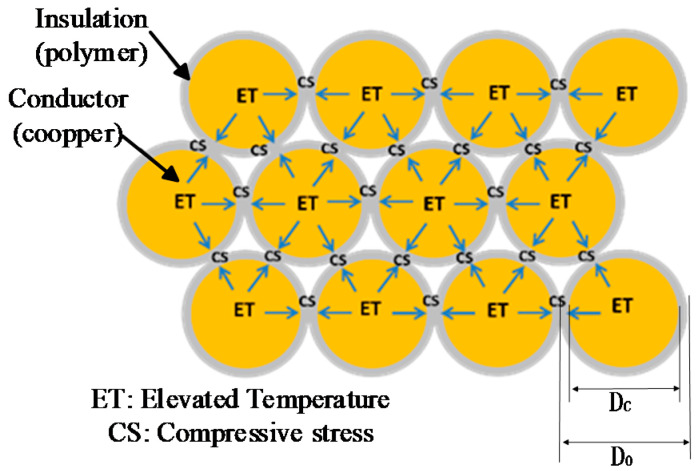
Cross-sectional view of a multilayer winding.

A cross-sectional view of an electromagnetic coil is shown in Figure 4. The magnet wire is constructed of conductor coated with insulation material. Excessive thermal stress is the primary cause that leads to insulation breakdown, especially for machines with safety-critical applications. For example, a solenoid valve (SOV) used in safety instrumented systems is maintained in an energized state throughout most of its installed life due to the adoption of the safety philosophy of returning to a fail-safe condition on the loss of air or electrical power. The consequence of the continuously energized SOVs is a more rapid degradation of the turn-to-turn insulation layers, which are usually composed of polymers, because of the long periods of thermal and compressive stresses resulting from Joule heating. These thermal and compressive stresses placed on the insulation create the necessary conditions for the occurrence of inter-turn insulation creep.

**Figure 5:**
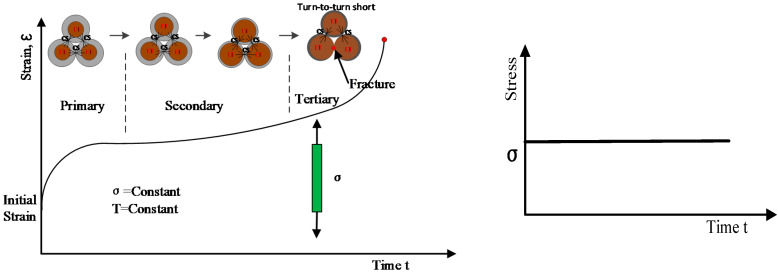
Idealized creep curve of plastics under constant temperature and constant load.

Figure 5 shows the idealized polymer curve for creep when both temperature and stress are constant [44]. The initial strain is roughly predicted by its stress-strain modulus. The material will continue to deform slowly with time indefinitely or until rupture or yielding causes failure. The primary region of the curve is the early stage of loading when the creep rate decreases rapidly with time. Then the creep rate reaches a steady state, which is called the secondary creep stage and is followed by a rapid increase (tertiary stage) and fracture [45].

According to the creep degradation mechanism analysis mentioned above, as shown in Figure 5, the inter-turn insulation layer continues to get thinner during the incipient insulation degradation phase. Finally, insulation fracture occurs, which leads to a turn-to-turn short, and the insulation deterioration process enters the late degradation phase. Thus, creep deformation of inter-turn insulation, which is a manifestation of insulation monitoring on the micro-level, provides a novel way for quantifying the insulation degradation status of coils in the incipient insulation degradation phase. Therefore, the insulation health indicator (*IHI*) can be defined from the creep degradation point of view. The related definitions are as follows.

(1) Insulation layer thickness (*ILT*):

(1) $$ILT=\left(D_{0}-D_{c}\right)/2$$

where $D_{0}$ is the outer radius and $D_{c}$ is the inner radius of the wire.

(2) Inter-turn insulation creep deformation (*CD*):

The creep deformation of inter-turn insulation is defined as the change of the inter-turn insulation layer thickness, which can be calculated as follows:

(2) $$CD=ILT_{\mathrm{healthy}}-ILT_{\mathrm{degraded}}$$

where $ILT_{\mathrm{healthy}}$ is the insulation layer thickness of the healthy coil and $ILT_{\mathrm{degraded}}$ is the insulation layer thickness of the degraded coil.

(3) Insulation health indicator (*IHI*):

(3) $$IHI=\frac{ILT_{\mathrm{degraded}}}{ILT_{\mathrm{healthy}}}*100\%$$

where 0≤IHI≤1.

### 2.2. Coil Electrical Behavior Analysis from a Creep Point of View

Coil electrical behavior is analyzed from a creep point of view in order to analyze the effect of inter-turn insulation creep degradation on coil high-frequency electrical parameter response. Equivalent circuit models (ECMs) can be used to analyze the electrical behavior of the electromagnetic coils. The ECM of the coil can be modeled as a resistance, *R*, and an inductance, *L*, in series, then parallel connected with a lumped capacitance, *C*. Since *R* and *L* are assumed to be constant during the coil incipient degradation phase, the evolution of *C* as the insulation degrades will be discussed in detail.

**Figure 6:**
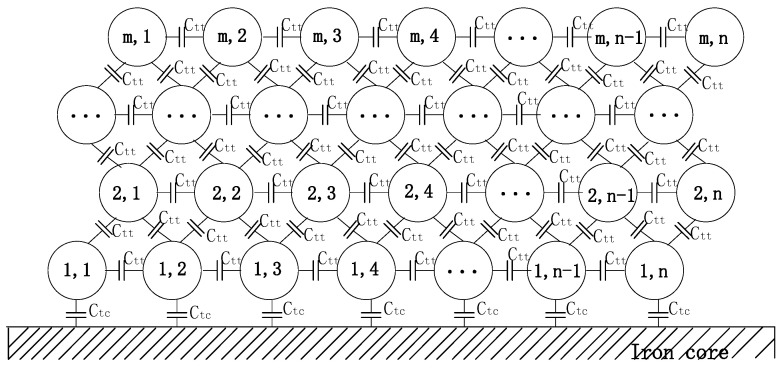
Stray capacitance network model of the electromagnetic coil with *m* layers and *n* turns per layer.

The parasitic capacitance network model, as shown in Figure 6, can be used to calculate the total parasitic capacitance of the coil, *C*, where $C_{tt}$ represents the turn-to-turn capacitance and $C_{tc}$ represents the capacitance between the iron core and innermost layer turn of the coil.

The turn-to-turn capacitance $C_{tt}$ can be calculated according to Equations (4) and (5) [46]:

(4) $$C_{tt}=\varepsilon_{0}l_{t}\left[\frac{\varepsilon_{r}\theta^{*}}{ln\frac{D_{0}}{D_{c}}}+\cot\left(\frac{\theta^{*}}{2}\right)-\cot\left(\frac{\pi }{12}\right)\right]$$

where

(5) $$\theta^{*}=\arccos\left(1-\frac{ln\frac{D_{0}}{D_{c}}}{\epsilon_{r}}\right)$$

where $D_{0}$ is the outer diameter of the magnetic wire including the insulation; $D_{c}$ is the inner diameter of the magnetic wire without the insulation (as shown in Figure 4); $l_{t}$ is the turn length; $\varepsilon_{r}$ is the relative permittivity of the insulation material; and $\varepsilon_{0}$ is the vacuum permittivity.

The capacitance between the iron core and innermost layer turn of the coil can be calculated according to Equation (6):

(6) $$C_{tc}=2C_{tt}$$

Multisim, a circuit simulation software, was used to find the relationship between *C* and $C_{tt}$ in [47], and the result is as follows:

(7) $$C=kC_{tt}$$

where *k* is an empirical constant that is dependent on the number of layers of the coil.

**Figure 7:**
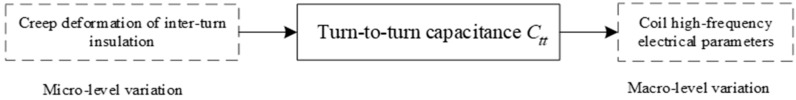
Effect of creep deformation of inter-turn insulation material on coil high-frequency parameters response.

According to theoretical derivation mentioned above, the effect of inter-turn creep deformation on coil high-frequency parameters response, such as coil parasitic capacitance *C*, can be analyzed, as shown in Figure 7. As the insulation degrades, inter-turn insulation creep deformation occurs on the micro-level. Then the inter-turn insulation creep deformation makes $D_{0}/D_{C}$ change, which leads to turn-to-turn capacitance $C_{tt}$ change. Finally, the coil high-frequency electrical parameter consequently changes as the $C_{tt}$ changes. Therefore, turn-to-turn capacitance $C_{tt}$ is the link between the micro-level and macro-level changes during the incipient insulation degradation process.

## 3. A Mapping Method from Coil Electrical Parameters to Inter-Turn Insulation Creep Deformation

Coil high-frequency electrical parameters can be used as on-line monitoring parameters during the incipient degradation phase because they are a manifestation of insulation degradation on the macro-level and are easy to measure in situ. A method is developed in this section to map coil high-frequency electrical parameters to inter-turn insulation creep deformation, which helps to determine the physical meaning of on-line monitoring parameters and thus presents opportunities to promote the accuracy of remaining useful life prediction of electromagnetic coil insulation.

**Figure 8:**
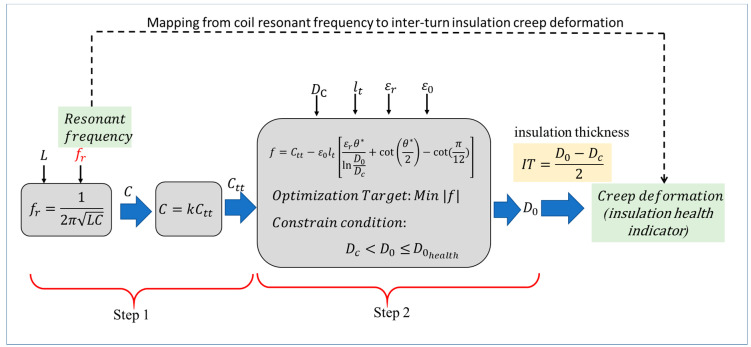
Framework for mapping from coil resonant frequency to inter-turn insulation creep deformation.

Resonant frequency is a commonly used parameter for coil health monitoring, and this section discusses the mapping method using coil resonant frequency as an example. Considering that $C_{tt}$ is the link between micro-level and macro-level variation during the insulation degradation process, a framework for mapping from coil resonant frequency to inter-turn insulation creep deformation is developed, which can be divided into the following 2 steps, as shown in Figure 8.

**(1) Step 1: Mapping from coil resonant frequency to turn-to-turn capacitance**

The resonant frequency, $f_{r}$, is defined as the frequency at which reactance is zero, and it can be obtained by splitting the impedance into its real (resistance) and imaginary (reactance) parts, then setting reactance equal to zero and solving for frequency. $f_{r}$ can be expressed as:

(8) $$f_{r}=\frac{1}{2\pi \sqrt{LC}}$$

where *L* is the coil inductance and *C* is the coil capacitance.

Then,

(9) $$C=1/(4\pi^{2}Lf_{r}^{2}k)$$

According to Equation (7),

(10) $$C_{tt}=1/(4\pi^{2}Lf_{r}^{2}k)$$

So, according to Equation (10), turn-to-turn capacitance $C_{tt}$ can be calculated by measuring the coil resonant frequency.

**(2) Step 2: Mapping from turn-to-turn capacitance to inter-turn insulation creep deformation**

Assume $D_{c}$ is constant during the incipient degradation phase, thus inter-turn insulation creep deformation can be obtained by calculating $D_{0}$ under the healthy and degraded cases, respectively. According to Equations (4) and (5), $D_{0}$ can be calculated by the following optimization method.

The optimization function:

(11) $$f=C_{tt}-\varepsilon_{0}l_{t}\left[\frac{\varepsilon_{r}\theta^{*}}{\ln\frac{D_{0}}{D_{c}}}+\cot\left(\frac{\theta^{*}}{2}\right)-\cot\left(\frac{\pi }{12}\right)\right]$$

where the optimization target is min |*f*|, and the constraint condition is $D_{C}<D_{0}<D_{0_{health}}$.

Since L, $D_{c}$, $l_{t}$, $\varepsilon_{r}$, and $\varepsilon_{0}$ can be obtained by measurement, $D_{0}$ can be calculated by the optimization process. Since it is a single variable minimization problem, optimization can be performed by a commercial solver tool. Then the inter-turn insulation creep deformation and the insulation health indicator can be calculated according to Equations (1) to (3).

Therefore, coil resonant frequency is mapped to the inter-turn insulation creep deformation by the two-step mapping.

## 4. Experimental Setup and Results

**Figure 9:**
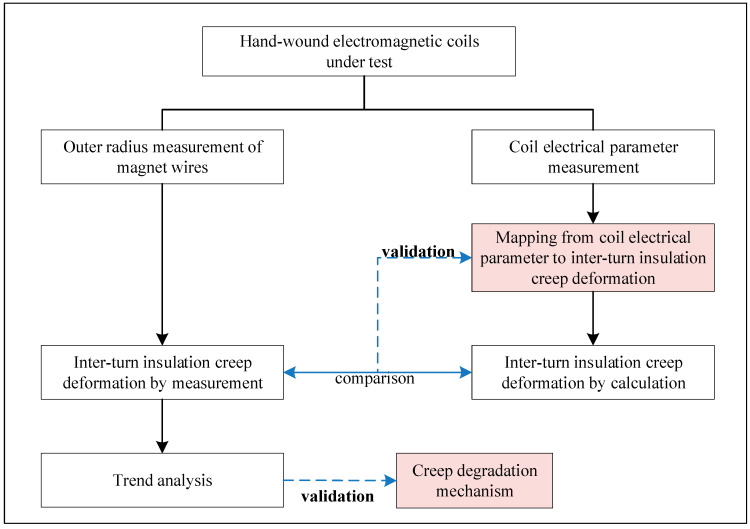
Illustration of the experimental process.

The purpose of the experiment was to validate the developed creep degradation mechanism and mapping method. The electromagnetic coil was hand-wound using the magnet wire, whose information is shown in Table 1. The coil was put into the chamber and removed periodically from the chamber every 15 h for measurement. As shown in Figure 9, both the coil electrical parameter and the outer radius of a magnet wire were measured after each accelerating cycle. First, a trend analysis of the magnet wire outer radius measurement results was used to validate the developed creep degradation mechanism. Second, the inter-turn insulation creep deformation by calculation based on coil electrical parameter and by actual measurement based on outer radius measurement results was compared to validate the developed mapping method.

**Figure 10:**
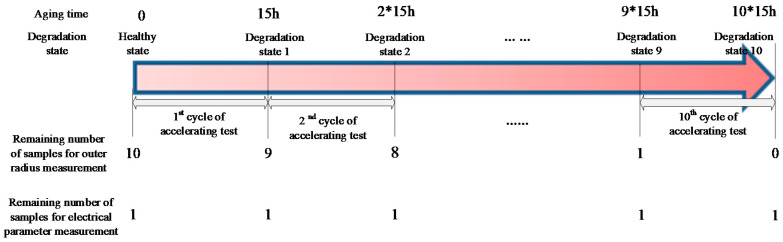
Description of the accelerated test process.

The experimental process is described in Figure 10. Ten cycles, namely, 150 h, of the thermally accelerated test were completed in total. Since the coil has to be unraveled in order to measure the outer radius of the magnet wire, the outer radius measurement was performed by destructive examination. Therefore, 10 coil samples were used for this type of measurement. After each accelerated test cycle, one coil was unraveled, and the outer radius of the magnet wire was measured. Then the sample was discarded. One additional sample was used for electrical parameter measurement. Further, the experimental process mentioned above was performed under the accelerated aging temperatures of 210 °C, 220 °C, and 230 °C, respectively. Therefore, (10 + 1) × 3 = 33 samples in total were used in this experiment.

### 4.1. Outer Radius Measurement of the Magnet Wires

**Figure 11:**
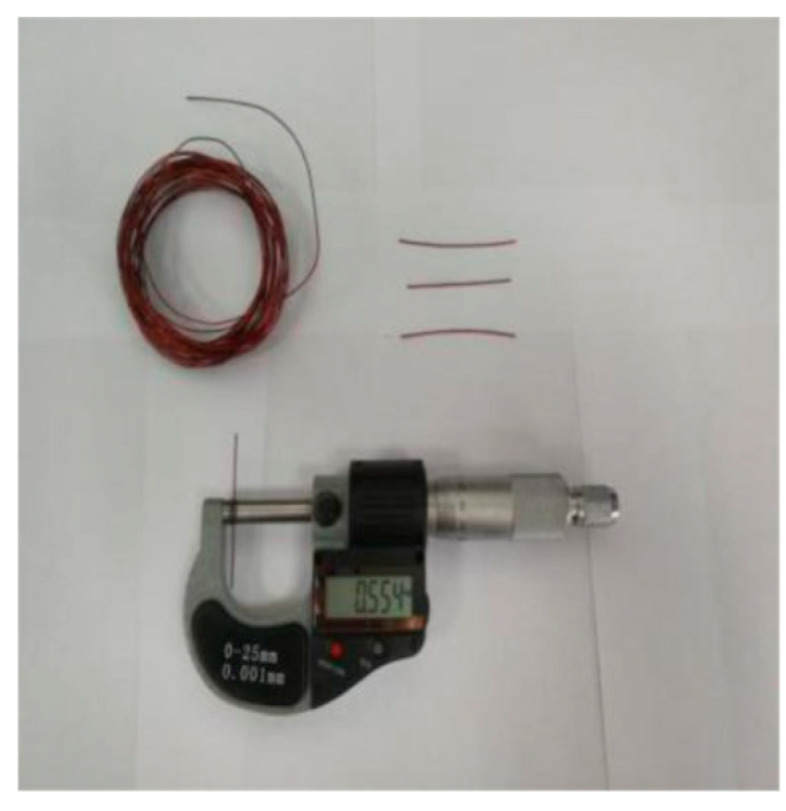
Experimental platform for magnet wire outer radius measurement.

**Figure 12:**
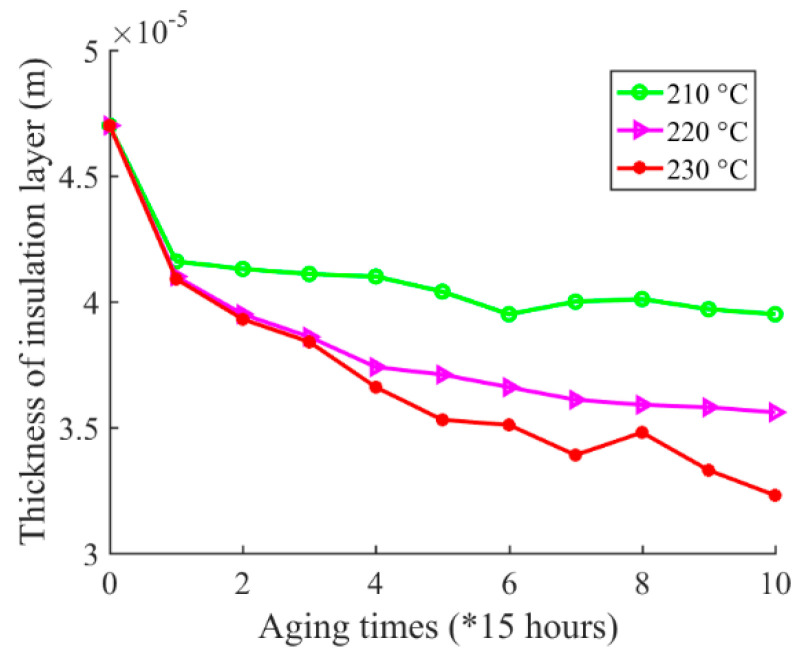
Magnet wire outer radius measurement results under 210 °C, 220 °C, and 230 °C.

**Figure 13:**
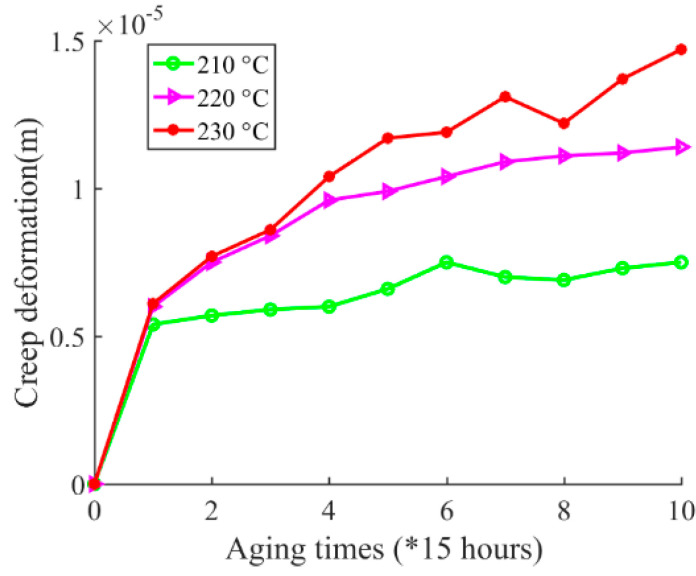
Inter-turn insulation creep deformations under 210 °C, 220 °C, and 230 °C.

The platform for outer radius measurement is shown in Figure 11. A micrometer was used to measure the outer radius of the magnet wire. The resolution ratio of the micrometer was 0.001 mm. The contact force between the micrometer screw and the sample under test was calibrated by measuring a healthy magnet wire. When the contact force was adjusted to 2 N, the measured value was 0.576 × 10^−3^ m, which is equal to the value by magnet wire specification as described in Table 1. Therefore, the contact force was fixed to 2 N in the following measurement during the experiment. Considering that the diameter of the wire may differ along the wire, as for each outer radius measurement after a certain accelerating cycle, the measurement is performed at 5 different positions along the length of wire in order to reduce the measurement uncertainties. Then the 5 measurement results are averaged, and the mean value is used as the final measurement value. The outer radius measurement results are shown in Figure 12. Further, according to Equation (2), the inter-turn insulation creep deformation can be calculated, and it has negative correlation to the outer radius of the wire. The inter-turn insulation creep deformations during the experiment are shown in Figure 13.

### 4.2. Coil Electrical Parameter Measurement

**Figure 14:**
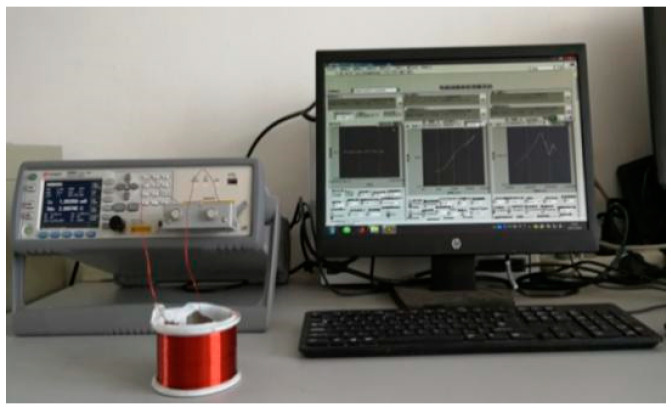
Impedance measurement platform for the hand-wound coil.

**Figure 15:**
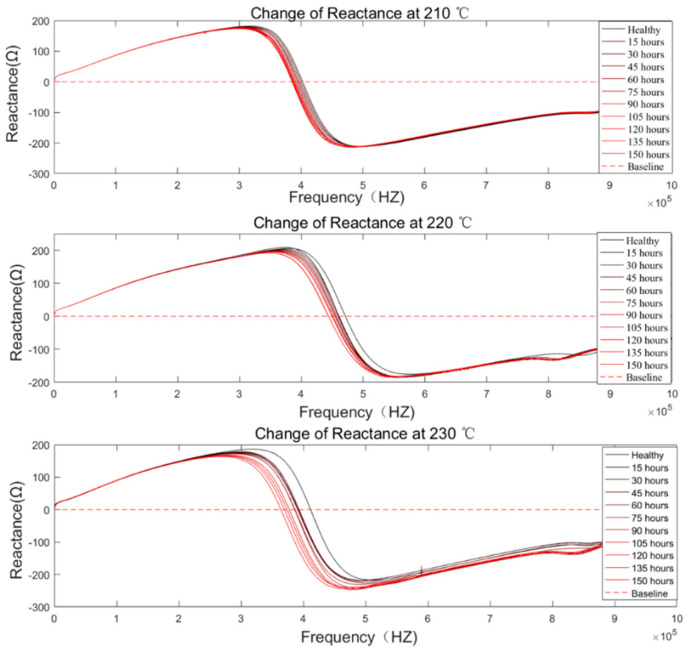
Reactance measurement results under 210 °C, 220 °C, and 230 °C.

**Figure 16:**
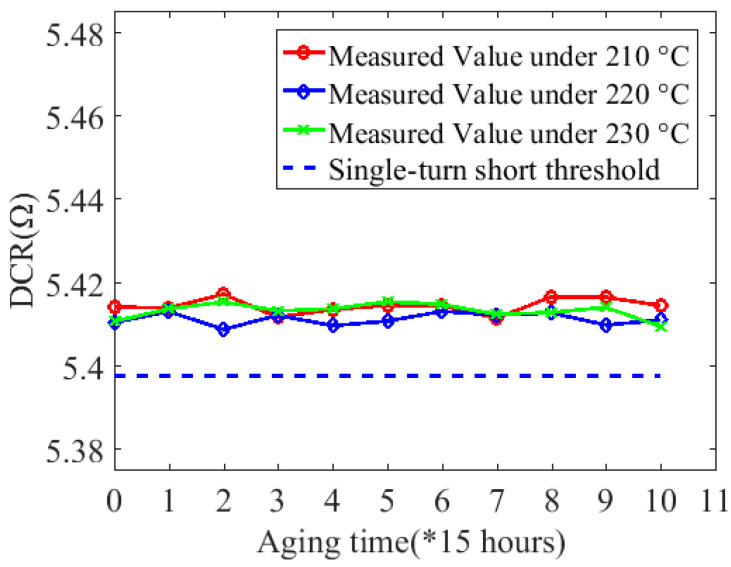
DCR measurement results under 210 °C, 220 °C, and 230 °C.

The coil electrical parameter measurement platform is shown in Figure 14. The terminals of the coil under test were connected to a Keysight E4980A LCR meter, and a 500-mV signal was applied across the coil. The impedance data collection by the LCR meter was controlled externally by LabVIEW and started only when the coil had cooled to room temperature. The reactance measurement results are shown in Figure 15. In addition, DC resistance (DCR) measurement was done at the same time to ensure the coil was in the incipient degradation phase during the experiment. The DCR measurement result is shown in Figure 16. Further, the DCR threshold for turn-to-turn short $DCR_{\mathrm{threshold}}$ is defined as:

(12) $$DCR_{\mathrm{threshold}}=DCR_{\mathrm{healthy}}-DCR_{\mathrm{singleturn}}$$

where $DCR_{\mathrm{health}}$ is 5.41 Ω according to Table 1, $DCR_{\sin\mathrm{gleturn}}$ refers to DCR of a single turn, and its measurement result is 0.0123 Ω.

**Figure 17:**
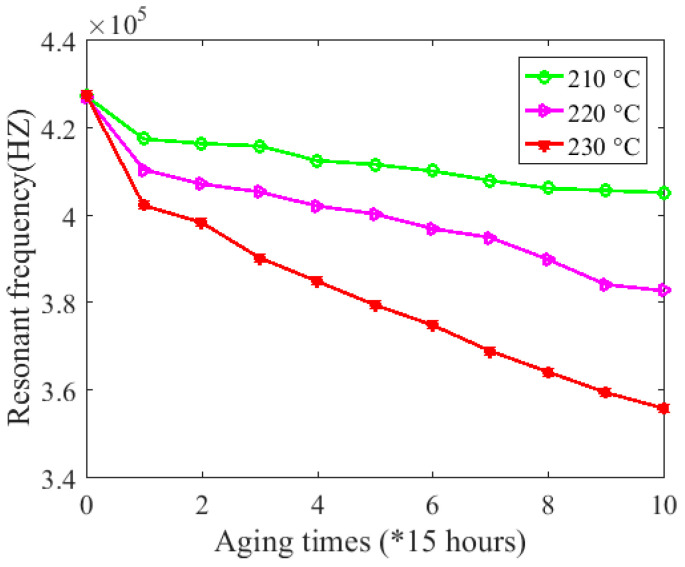
Relationship between resonant frequency and aging time under 210 °C, 220 °C, and 230 °C.

Thus, the calculated result for $DCR_{\mathrm{threshold}}$ is 5.3977 Ω. As shown in Figure 16, all 10 DCRs measured were greater than $DCR_{\mathrm{threshold}}$, which means that the coils under test were all in the incipient degradation phase during the 10 accelerated cycles. Finally, the relationship between resonant frequency and aging time under these three temperatures can be obtained, as shown in Figure 17.

**Figure 18:**
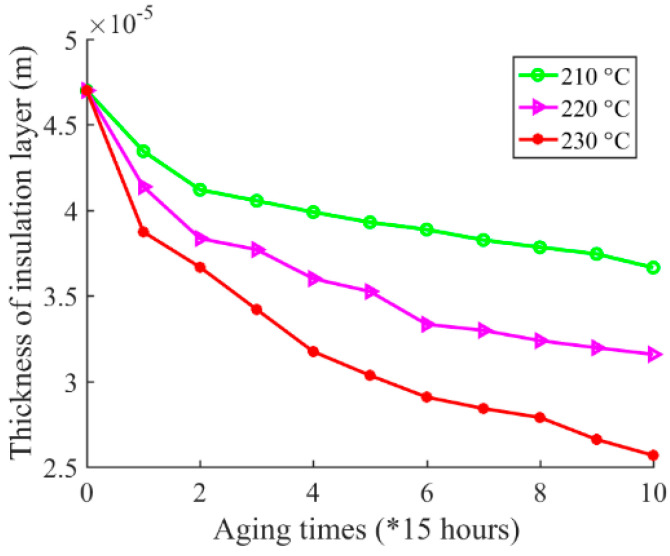
Insulation thickness by calculation based on coil high-frequency electrical parameter under 210 °C, 220 °C, and 230 °C.

**Figure 19:**
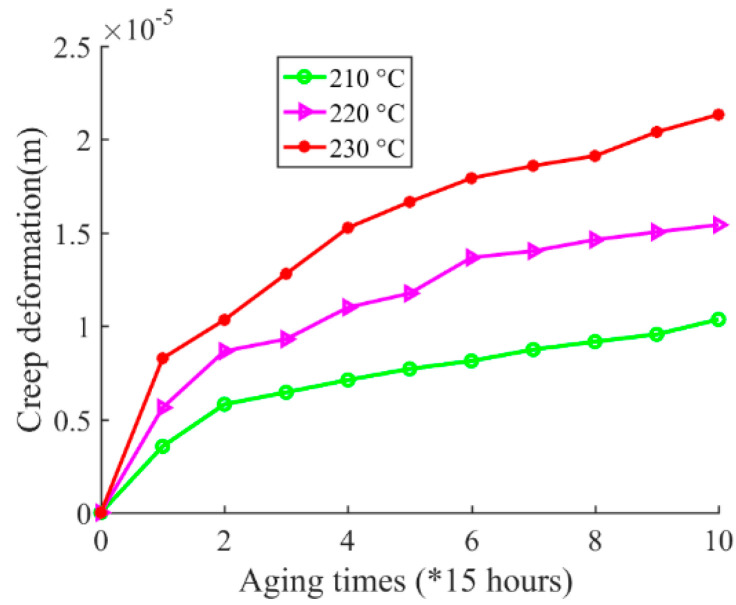
Inter-turn insulation creep deformation by calculation based on coil high-frequency parameter under 210 °C, 220 °C, and 230 °C.

Next, coil resonant frequency is mapped to inter-turn insulation creep deformation by the mapping method developed in Section 3. The hand-wound coil parameters of $l_{t}$, $\varepsilon_{r}$, $\varepsilon_{0}$, $D_{c}$, L are shown in Table 1. $D_{0}$ can be calculated according to optimization function Equation (11), which is implemented by a commercial solver tool. Inter-turn insulation thickness and creep deformation can consequently be calculated according to Equations (1) and (2). Thus, the curve fitting of the relationship between inter-turn insulation thickness and aging time, and the relationship between inter-turn insulation creep deformation and aging time, are shown in Figure 18 and Figure 19, respectively.

### 4.3. Experimental Result Analysis

First, according to the magnet wire outer radius measurement results, as shown in Figure 12 and Figure 13, the inter-turn insulation thickness showed a decreasing trend while the inter-turn insulation creep deformation had an increasing trend in general during the experiment. However, the data do not show a monotonous decreasing or increasing trend. The reason for this is that there are differences in temperature at different positions inside the chamber. For example, there is a higher temperature near the heater, whereas there is a lower temperature near the air vent of the chamber. As mentioned earlier, 10 samples were placed in the chamber for magnet wire outer radius measurement under each specified accelerated aging temperature at the beginning of the test. The temperature difference at the different locations inside the chamber leads to the appearance of outlier data points during the experiment, such as the data point at the 6th cycle under 210 °C and the data point at the 8th cycle under 230 °C. Further, as the accelerated aging temperature increased, the creep rate increased. These trends validate the developed creep degradation mechanism.

**Figure 20:**
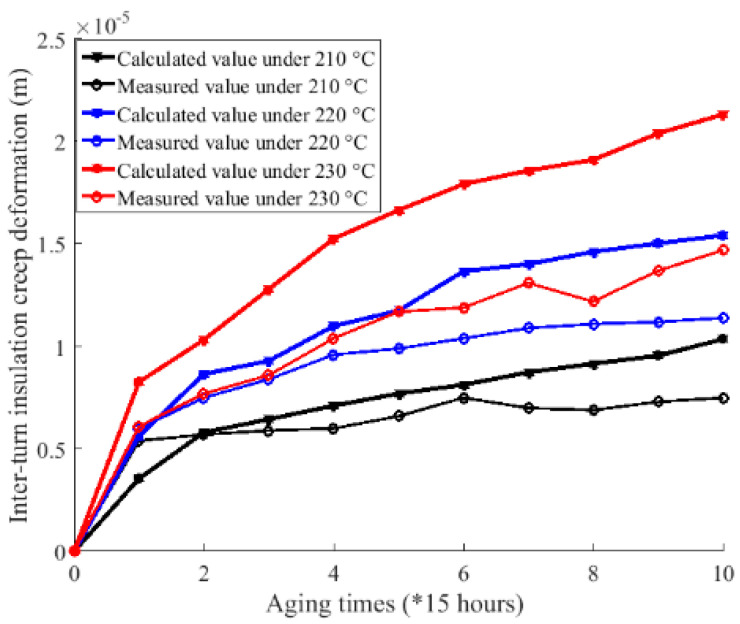
Comparison between calculated and measured creep deformation under 210 °C, 220 °C, and 230 °C.

**Figure 21:**
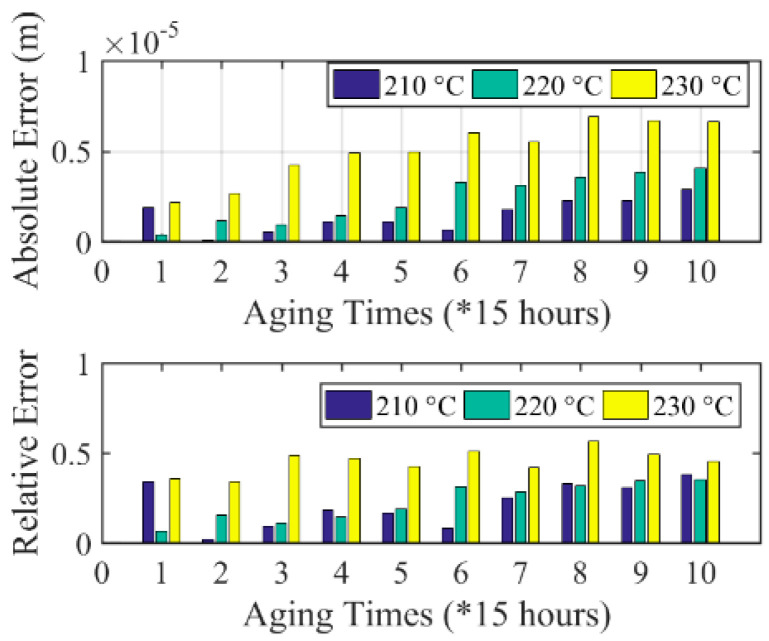
Errors between calculated and measured creep deformation under 210 °C, 220 °C, and 230 °C.

Second, the measured and calculated results of inter-turn insulation creep deformation, as shown in Figure 13 and Figure 19, respectively, are merged to Figure 20 for comparison. Further, the absolute and relative errors between calculated and measured results are shown in Figure 21. It can be seen from Figure 20 that the calculated creep deformation exceeds the measured creep deformation in general under each accelerating temperature. The reason for this is that the sample used for electrical parameter measurement is placed near the heater of the chamber, which makes the accelerating temperature for the sample used for electrical parameter measurement higher than the samples used for the magnet wire outer radius measurement. Further, as shown in Figure 21, the comparison results show that the errors between calculated and measured inter-turn insulation results are acceptable, which validates the developed mapping method.

**Figure 22:**
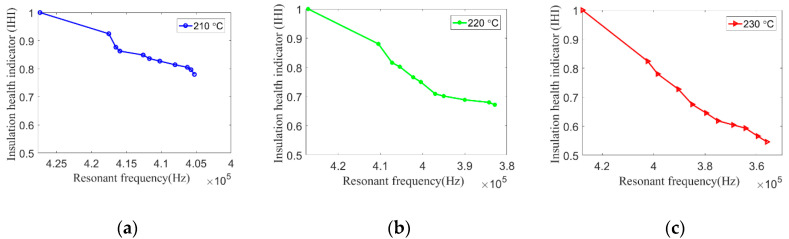
Relationship between insulation health indicator (*IHI*) and resonant frequency under (**a**) 210 °C, (**b**) 220 °C, and (**c**) 230 °C.

**Figure 23:**
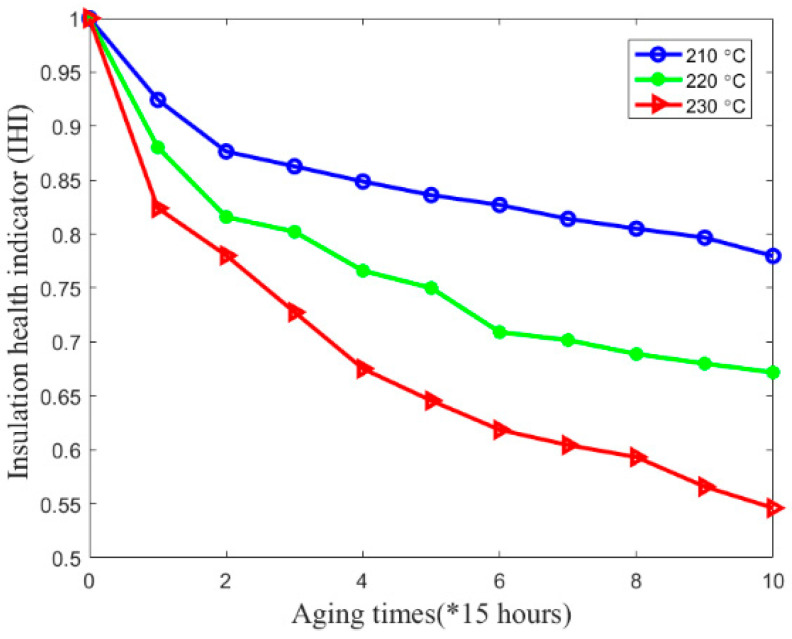
Relationship between *IHI* and aging time under 210 °C, 220 °C, and 230 °C.

Finally, coil resonant frequency is used to calculate inter-turn insulation creep deformation and *IHI* by the developed mapping method. As shown in Figure 17, coil resonant frequency showed a decreasing trend during the experiment. However, the relationship between resonant frequency and coil health status is still not clear. Therefore, according to the proposed mapping method and Equations (1)–(3), the correlation between coil resonant frequency and *IHI* under different aging temperatures can be explored, as shown in Figure 22a–c. The relationship between *IHI* and aging time can thus be obtained, as shown in Figure 23. Since coil resonant frequency can be measured in situ, this makes sense for improving the performance of insulation degradation monitoring based on coil high-frequency electrical parameters. For example, the failure threshold of coil resonant frequency can be set according to its corresponding *IHI*.

## 5. Conclusions

Electromagnetic coils are widely used components in many applications and systems, including solenoids, motors, and transformers. However, coil insulation systems are failure-prone, especially under excessive thermal stresses. Further, the coil insulation degradation process accelerates once a turn-to-turn short has occurred, and the impending insulation failure usually occurs suddenly and has catastrophic effects. Thus, insulation degradation monitoring prior to the formation of turn-to-turn shorts presents opportunities for decreasing the safety risk and economic loss due to the unexpected shutdown of machines that incorporate electromagnetic coils.

This paper investigated degradation monitoring of coil insulation systems under thermal loading conditions, which is the primary factor that leads to insulation breakdown, from a creep point of view. Polymer creep curves obtained by inter-turn insulation degradation mechanism analysis and outer radius measurement results of magnet wires during accelerated tests show that the insulation degradation process can be quantified by inter-turn insulation creep deformation on a micro-level. Thus, the insulation health indicator was defined based on insulation layer thickness.

A method of mapping coil high-frequency electrical parameters to inter-turn insulation creep deformation was explored by circuit theories and optimization methods, which is validated by comparison between mapped and measured creep deformation during the thermally accelerated tests. The developed method bridges the gap between the micro-level and macro-level changes during the incipient insulation degradation process, and a knowledge base for insulation degradation monitoring can thus be established by converting the electrical monitoring parameter to coil insulation health status. The developed mapping method helps to reveal the practical physical meaning of coil high-frequency electrical parameters and enhance the prognostic ability of existing high-frequency electrical parameter-based insulation degradation monitoring methods.

## Figures and Tables

**Table 1:** Specimen information.

Parameter Properties	Numerical Value
$DCR_{\mathrm{healthy}}$ (Ω)	5.41
Magnet wire insulation class	Class B
Insulation composition	Polyester
$l_{t}$ (m)	2.65 × 10^−1^
$\varepsilon_{r}$	3.5
$\varepsilon_{0}$	8.8541878 × 10^−12^
$D_{0}$ (m)	0.576 × 10^−3^
L (H)	0.001075
$D_{C}$ (m)	0.482 × 10^−3^
